# Comparison Between Intravitreal Anti-Vascular Endothelial Growth Factor Monotherapy and Vitrectomy in Age-Related Macular Degeneration with Large Submacular Hemorrhages

**DOI:** 10.3390/jcm14051477

**Published:** 2025-02-22

**Authors:** Misa Miyazato, Maiko Maruyama-Inoue, Shin Tanaka, Tatsuya Inoue, Yasuo Yanagi, Kazuaki Kadonosono

**Affiliations:** Department of Ophthalmology, Yokohama City University Medical Center, Yokohama, Japan 4-57 Urafune-cho, Minami-ku, Yokohama 232-0024, Kanagawa, Japan; miyazatomisa@gmail.com (M.M.); tanaka_s@yokohama-cu.ac.jp (S.T.); inouet@yokohama-cu.ac.jp (T.I.); yasuo.yanagi.tokyo@gmail.com (Y.Y.); kado@med.yokohama-cu.ac.jp (K.K.)

**Keywords:** age-related macular degeneration, submacular hemorrhage, vascular endothelial growth factor, brolucizumab, aflibercept, vitrectomy

## Abstract

**Objectives:** To compare the 1-year visual outcomes of patients treated with intravitreal anti-vascular endothelial growth factor (VEGF) monotherapy or vitrectomy for large submacular hemorrhages (SMHs) due to neovascular age-related macular degeneration (nAMD). **Methods:** We retrospectively studied 31 eyes with severe SMHs exceeding 3 disc areas (DAs) secondary to nAMD treated with anti-VEGF agents or a vitrectomy. Patients undergoing anti-VEGF monotherapy received three monthly loading doses of intravitreal injections of aflibercept or brolucizumab followed by as-needed injections or proactive treatment (anti-VEGF group); those undergoing vitrectomies underwent a 25-gauge vitrectomy and a submacular injection of tissue plasminogen activator (25 μg) and 0.4 mL of air with a microneedle having an outer diameter of 50 μm. The best-corrected visual acuities (BCVAs) were compared before and 6 and 12 months after initial treatment. Factors affecting the visual acuity (VA) at 12 months and VA improvements were determined. **Results:** A total of 17 eyes from 16 patients (54.8%) received anti-VEGF treatment and 14 eyes from 14 patients (45.2%) underwent vitrectomy. The baseline and 12-month mean logarithm of the minimum angle of resolution BCVAs in all eyes after treatment were 0.78 and 0.82, respectively, which were not significantly different (*p* = 0.661). The lens status, central foveal thickness (CFT) height, and baseline VA were associated significantly with the 12-month BCVA (*p* = 0.028, *p* = 0.008, and *p* = 0.021, respectively) and VA improvement (*p* = 0.015, *p* = 0.002, and *p* = 0.003, respectively). **Conclusions:** Anti-VEGF monotherapy and vitrectomy maintained functionality in patients with large SMHs due to nAMD. Greater CFT was associated with worse 12-month BCVA and less BCVA improvement despite the treatment modality.

## 1. Introduction

Untreated neovascular age-related macular degeneration (nAMD) is a leading cause of irreversible visual impairment in developed countries [1,2]. In particular, if submacular hemorrhages (SMHs) occur, the combination of a diffusion hemorrhagic barrier, mechanical damage to the photoreceptors by hemorrhagic contraction, and iron toxicity leads to a poor visual prognosis [3,4]. Therefore, early therapeutic intervention in patients with a SMH secondary to nAMD is necessary. Previous reports have described that SMHs secondary to nAMD have been treated by pneumatic displacement (PD) [5], a vitrectomy with gas tamponade [6], utilization of a tissue plasminogen activator (tPA) [7], anti-vascular endothelial growth factor (VEGF) agents injection [8,9], and the combining of these treatments. However, controversy remains regarding which treatment modality should be selected to treat nAMD with SMHs.

We previously reported that anti-VEGF monotherapy was well tolerated in SMH patients due to nAMD unless vitreous hemorrhages (VHs) occurred during the follow-up [9]. SMHs with larger areas especially were related with the higher possibility of a VH occurring, which resulted in poorer visual prognosis [9]. However, our group also reported that vitreous surgery combined with tPA and air improved vision in 84.6% of patients with nAMD with SMHs [6]. Previous reports have compared these treatment modalities using meta-analysis or a multicenter study and reported that both anti-VEGF monotherapy and surgery in patients with SMHs due to nAMD showed similar visual acuity (VA) outcomes [10,11]. However, those two treatment modalities have not been compared at one institution, while the choice between anti-VEGF monotherapy and vitrectomy to treat SMHs due to nAMD is crucial. The present study compared the 1-year functional outcomes of anti-VEGF monotherapy and vitrectomies in patients with large SMHs due to nAMD in routine clinical practice. The factors affecting the visual outcomes also were investigated in patients with SMHs secondary to nAMD.

## 2. Materials and Methods

We retrospectively studied 31 consecutive eyes with severe SMHs secondary to nAMD that were treated with anti-VEGF monotherapy (Aflibercept, Eylea, Bayer Health Care, Berlin, Germany, or brolucizumab (Brolucizumab, Beovue, Novartis Pharmaceuticals, Basel, Switzerland) or a 25-gauge vitrectomy combined with a tPA and air injection. Two retina specialists (M.M.I. and K.K.) independently assigned the patients to a treatment. Treatment modalities were decided comprehensively based on baseline VA, age, the size of the SMH, and spectral-domain optical coherence tomography (SD-OCT) findings. All patients received initial treatment at Yokohama City University Medical Center between June 2019 and June 2023. This study was approved by the institutional review board of the Yokohama City University Medical Center and was conducted according to the tenets of the Declaration of Helsinki. Written informed consent was provided by all patients before their medical record data were collected for this research.

The inclusion criteria included a VA of 20/40 or less at the time of the initial anti-VEGF injection or surgery and the presence of a SMH exceeding 3 disc areas (DAs) secondary to nAMD that included the fovea and were determined by clinical findings. The patients included in the study all had images available that were gained by fluorescein angiography, indocyanine green angiography (Spectralis Product Family Version 5.3), and SD-OCT (Spectralis Product Family Version 5.3, Heidelberg Engineering, Heidelberg, Germany). Patients were excluded who had a history of ocular diseases such as uncontrolled glaucoma, uveitis, retinal vein occlusion, and rhegmatogenous retinal detachment (RRD). Patients who had a VH before the initial treatment were also excluded.

All patients treated with anti-VEGF monotherapy (anti-VEGF group) were treatment-naïve and received three consecutive monthly intravitreal injections of aflibercept (IVA) or brolucizumab (IVBr) during the loading phase. For patients who received IVA, in the maintenance phase, the injection was performed based on an as-needed or treat-and-extend regimen. Patients who received IVBr were treated every 12 weeks unless new fluid or a new hemorrhage developed. If that occurred, the treatment interval was shortened to 8 weeks. If a VH developed after the treatment, anti-VEGF treatments were discontinued, and vitrectomy surgery was performed.

Patients, including those with previous anti-VEGF injections, underwent a 25-gauge vitrectomy using the Constellation Vision System (Alcon, Fort Worth, TX, USA). A total of 7 out of the 14 patients (50%) who had phakic all received a pars plana vitrectomy combined with cataract surgery. tPA (Monteplase; Eisai Co., Ltd., Tokyo, Japan) was dissolved in saline and adjusted to 80,000 units/mL. After core vitrectomy and peripheral vitreous shaving, tPA was injected slowly into the subretinal space using a microneedle with an outer diameter of 50 μm (Tochigi Seiko, Co., Ltd., Tochigi, Japan). Then, approximately 0.4 mL of air was injected into the subretinal space using the same microneedle at a pressure of 4 to 6 psi to move the dissolved clot as previously described [6]. Postoperatively, patients were placed in a head-upright position for 1 day so that the dissolved clot could move outside the macula via air pressure. If a VH developed postoperatively, vitrectomy was performed again. Also, anti-VEGF agents were administered as needed during or after the surgery.

The primary outcome was the changes in the best-corrected VA (BCVA) in each group. The secondary outcomes were the determination of the factors affecting the visual outcomes in patients with SMHs secondary to nAMD. Multiple regression analyses were performed to identify correlations between the parameters, including age, sex, lens status (phakia or intraocular lens [IOL]), treatment modality (anti-VEGF or vitrectomy), use or no use of anticoagulants, symptom duration, DAs, baseline BCVA, baseline central foveal thickness (CFT), baseline SMH thickness at the fovea, baseline hemorrhagic pigment epithelial detachment (PED) thickness at the fovea, post-injection BCVA at 12 months, and the BCVA changes. The post-injection 12-month BCVA and the changes in the BCVA were used as dependent variables.

The BCVAs before and after treatment were compared using one-way analysis of variance with Bonferroni correction. The baseline characteristics were compared between the anti-VEGF and vitrectomy groups using unpaired *t* test and the Fisher’s exact test. The proportion of the patients who developed a VH during the follow-up period between the two groups was compared using the Fisher’s exact test. The statistical analysis software used was Ekuseru-Toukei 2012 (Social Survey Research Information, Tokyo, Japan). *p* < 0.05 was considered significant.

## 3. Results

A total of 31 eyes from 31 patients (24 men, 7 women; mean age, 73.4 ± 9.3 years; range, 48–95 years) had a large SMH that exceeded 3 DAs due to nAMD and were assessed at the 12-month follow-up examination. Each eye analyzed in the study was subjected to its first SMH. The baseline characteristics and clinical data for each patient before treatment are shown in Table 1. A total of 17 eyes were treated with anti-VEGF monotherapy and the remaining 14 eyes were treated with vitrectomies. All patients in the anti-VEGF group were treatment-naïve, while 4 of the 14 patients (28.6%) in the vitrectomy group had received anti-VEGF treatment before the initial treatment. In patients in the anti-VEGF group, 8 patients were treated with aflibercept and 9 patients were treated with brolucizumab. The mean number of injections in the anti-VEGF group was 5.3 ± 1.6 during the follow-up period. Meanwhile, 10 of the 14 patients (71.4%) in the vitrectomy group received aflibercept injections during or after the surgery. The mean number of injections in the vitrectomy group was 3.9 ± 2.8 during the follow-up period. The vitrectomy group had significantly more patients with IOLs, worse baseline BCVAs, larger CFTs, and larger SMH thicknesses (*p* = 0.004, *p* = 0.004, *p* = 0.009, and *p* = 0.025, respectively).

### 3.1. Changes in the BCVAs

**Figure 1:**
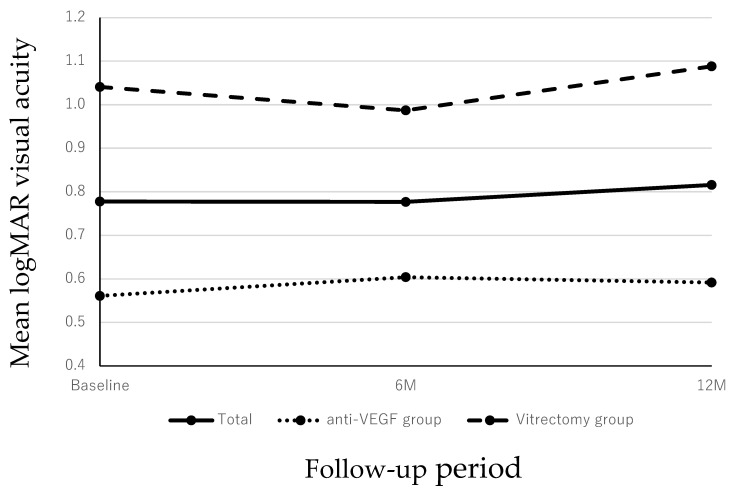
Changes in the BCVA during the 12-month follow-up period. In all cases, the mean BCVA at 6 and 12 months had not improved significantly compared with the preoperative VA (*p* = 0.999 and *p* = 0.999 at 6 and 12 months, respectively). In both patients treated with anti-VEGF monotherapy or vitrectomy, the post-injection BCVA did not improve significantly compared with baseline throughout the 12-month period (*p =* 0.999 and *p =* 0.999 at 6 and 12 months, respectively for both groups).

In all patients, the baseline mean logarithm for the minimum angle of resolution (logMAR) BCVA was 0.78 ± 0.48. The mean logMAR BCVAs at 6 and 12 months after the initial treatment, respectively, were 0.78 ± 0.48 and 0.82 ± 0.57, which did not differ significantly compared with the baseline (both *p* > 0.05 at 6 and 12 months). Meanwhile, the mean logMAR BCVAs at baseline were 0.56 ± 0.27 in the anti-VEGF group and 1.04 ± 0.55 in the vitrectomy group. The mean logMAR BCVAs at 6 and 12 months in both groups after the initial treatment, respectively, were 0.60 ± 0.52 and 0.59 ± 0.52 in the anti-VEGF group and 0.99 ± 0.56 and 1.09 ± 0.52 in the vitrectomy group. In both groups, the postoperative BCVA did not improve significantly compared with the preoperative status (*p* > 0.05 in both groups) (Figure 1).

### 3.2. Factors Affecting the BCVA at 12 Months and the BCVA Improvement

**Figure 2:**
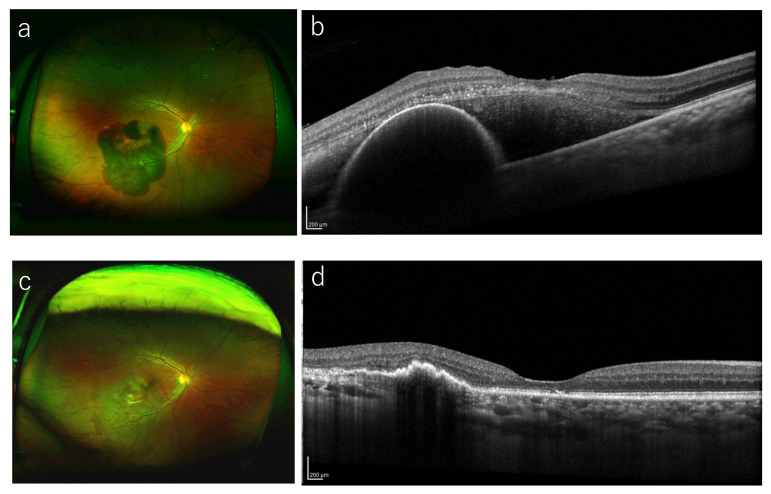
A representative case treated with anti-VEGF monotherapy. (**a**) A 63-year-old man presented with visual loss in his left eye (BCVA 20/40). Funduscopic examination shows a large SMH including the macula. (**b**) A baseline OCT image shows the SMH with thickness of 454 μm. He started intravitreal injection of brolucizumab. (**c**) Twelve months after the initial treatment, the SMH resolved completely and his BCVA improved to 20/16. (**d**) OCT at 12 months shows the improvement of SMH and hemorrhagic PED.

**Figure 3:**
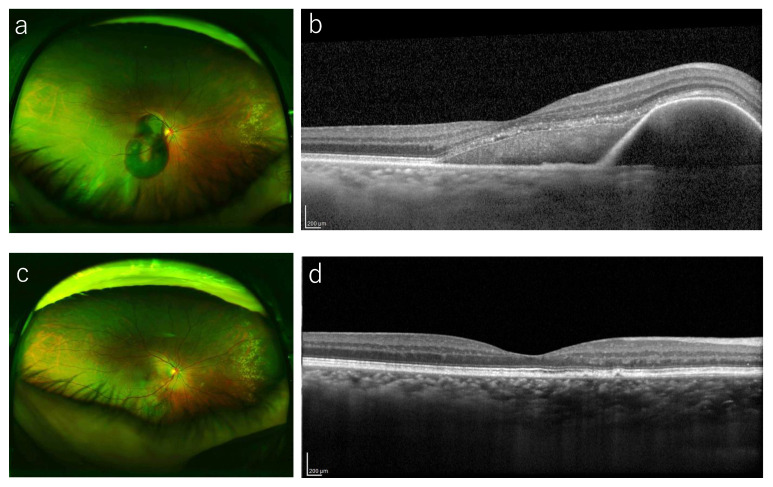
A representative case treated with vitrectomy. (**a**) A 72-year-old woman presented with sudden visual loss in her right eye (BCVA 20/40). Funduscopic examination in that eye shows a severe SMH in the macula. (**b**) OCT at baseline shows a SMH with thickness of 401 μm. A 25-gauge vitrectomy with tPA and gas injection was performed. (**c**) A fundus photography at 12 months shows that the SMH resolved. (**d**) OCT at 1 year shows improvement of the SMH and hemorrhagic PED.

Multiple linear regression analysis indicated that the lens status, CFT height, and the baseline VA were associated significantly with the 12-month BCVA (*p* = 0.028, *p* = 0.008, and *p* = 0.021, respectively) (Table 2). That is, phakia, lower CFT, and better baseline BCVA tended to be associated with better BCVA at 12 months. These factors also were associated significantly with the BCVA improvement (*p* = 0.015, *p* = 0.002, and *p* = 0.003, respectively). Phakia, lower CFT, and worse baseline BCVA tended to be associated with larger BCVA improvement at 12 months. No significant correlations were observed between age, sex, use or no use of anticoagulants, treatment modality (anti-VEGF or vitrectomy), symptom duration, DAs, SMH thickness at the fovea, hemorrhagic PED thickness at the fovea, 12-month BCVA, or the changes in the BCVA (*p* > 0.05 for all comparisons). Figure 2 and Figure 3 show representative cases in the anti-VEGF group and vitrectomy groups, respectively.

### 3.3. Complications During the Follow-Up Period

A VH developed after the initial treatment in two eyes (11.8%) in the anti-VEGF group and four eyes (28.6%) in vitrectomy group, which did not differ significantly (*p* = 0.370). All cases underwent vitrectomy after the VH developed. No other surgical complications, such as macular hole formation or RRD, occurred during the follow-up period. Two eyes (14.3%) in the vitrectomy group had a recurrent SMH that was controlled using additional anti-VEGF injections. By contrast, one eye (5.9%) in the anti-VEGF group had a retinal pigment epithelial tear after first brolucizumab injection.

## 4. Discussion

The current study has shown that both anti-VEGF injections and vitrectomy maintained the VA in patients with large SMHs secondary to nAMD. However, patients with a larger pre-treatment CFT had a poorer visual prognosis regardless of the treatment modality used.

Currently, several studies have compared treatment modalities in patients with SMH due to nAMD. Sim et al. compared PD combined with anti-VEGF therapy and anti-VEGF monotherapy, and the visual outcomes at 12 months were poor regardless of the treatment modality [12]. Barzelay et al. reported that vitrectomy with tPA was associated with better VA outcomes compared with PD with tPA for small and medium SMHs [13]. Meanwhile, Gabrielle et al. reported that vitrectomy did not achieve better visual gains for SMHs due to nAMD compared with PD at 3 months, with anti-VEGF added to each arm [14]. However, no reports have compared anti-VEGF injections and surgery at the same institution. As a result, in our study, the treatment modalities were not associated with visual outcomes at 12 months and the VA improvement.

Previous studies have reported that anti-VEGF monotherapy and vitrectomy were both effective for SMHs secondary to nAMD. Kim et al. treated 49 eyes with SMH due to nAMD using anti-VEGF agents (Ranibizumab, Lucentis, Genentech, South San Francisco, CA; or Bevacizumab, Avastin, Genentech) and reported that 49% of patients achieved more than three lines of visual improvement [15]. Kamei and Tano reported that the BCVA improved in 83% of eyes during a mean follow-up time of 6.9 years in patients with SMHs treated with tPA-assisted vitrectomies [16]. However, our study showed that the VA did not improve during the follow-up period in patients with large SMHs due to nAMD treated with either anti-VEGF agents or a vitrectomy. We speculated that this might have resulted from patient selection. To compare these treatment modalities, we specifically targeted patients with relatively severe SMHs with baseline VAs of 20/40 or less and SMHs exceeding 3 DAs. Therefore, these patients already may have had irreversible retinal damage caused by iron and hemosiderin toxicity to the retina, metabolic disturbances between the outer retina and retinal pigment epithelium, and traction of fibrin clots, which resulted in no VA improvement. However, the baseline VA was associated significantly with the 12-month VA. This means that patients with SMHs due to nAMD should be treated as early as possible regardless of the treatment modalities.

Furthermore, in our study, phakia also was associated significantly with better VA and 12-month visual improvement. This might have resulted from cataract surgery, which was performed simultaneously when the vitrectomies for the SMHs were performed in the vitrectomy group. In addition, the CFT height was also related to the visual prognosis regardless of the treatment modality, which is consistent with previous reports [15,17]. Therefore, physicians should especially consider that higher CFTs are risk factors for worse VAs.

In this study, 11.8% (two eyes) in the anti-VEGF group and 28.6% (four eyes) in the vitrectomy group developed VHs. Although there was no significant difference in the proportion of VHs after the initial treatment between the two groups, the VHs tended to develop in the vitrectomy group during the follow-up. A reason for this might be due to the baseline differences between the two groups. Although we targeted large SMHs due to nAMD of 3 DAs or more, the vitrectomy group had a worse baseline VA, higher CFTs, and higher SMHs at baseline, which caused bias between the two groups. Another reason is that 28.6% of eyes in the vitrectomy group had already been treated with anti-VEGF injections; therefore, these cases might have had difficult-to-control nAMD using anti-VEGF treatment monotherapy. Furthermore, even during or after the surgery, 71.4% of patients received additional anti-VEGF agents, meaning that the nAMD activity remained after the initial surgery. Therefore, it cannot be said that the vitrectomy group was more likely to develop VHs as a treatment complication, rather the VHs could result from the nAMD activity after the initial vitrectomy.

The limitations of this study were its retrospective nature and small sample size, which can introduce biases related to patient selection and data collection and may affect the reliability of the outcomes reported. It is necessary to perform a large-scale randomized study to confirm these current results. Another limitation was the significant differences in the baseline characteristics between the two groups, which means that the vitrectomy group still included more severe cases than the anti-VEGF and made it difficult to draw direct comparisons with the anti-VEGF group. However, the use of multiple regression analysis in our study showed that both the 12-month VA and the visual improvement were not associated with the treatment modalities. Therefore, we believe that both treatments were well tolerated to prevent VA decline in patients with large SMHs secondary to nAMD. Also, some patients in the vitrectomy group had previously received anti-VEGF injections, which may have influenced their response to the vitrectomy procedure. This prior treatment could complicate the assessment of the effectiveness of vitrectomy alone. Therefore, it is necessary to perform this study under more similar baseline conditions of the two groups in the future.

In conclusion, both anti-VEGF monotherapy and vitrectomy effectively maintained the VA for 1 year in patients with large SMHs due to nAMD. Each treatment modality has its own characteristics. Anti-VEGF treatment is less invasive. For elderly patients who have difficulty being hospitalized for a long time or maintaining a face-down position, it may be worthwhile to administer anti-VEGF drugs first. However, a vitrectomy may be more appropriate for patients who have problems with repeated numbers of treatments or high cost or for the most severe cases. However, physicians should be cautioned that higher CFTs at baseline could result in unfavorable visual outcomes regardless of the treatment modality.

## Figures and Tables

**Table 1:** Comparison between anti-VEGF group and vitrectomy group.

	Total (n = 31)	Anti-VEGF Group (n = 17)	Vitrectomy Group(n = 14)	*p*-Value *
Number of patients	31	17	14	
Sex (Male/Female)	24/7	15/2	9/5	0.198
Age, mean ± SD, year (range)	73.4 ± 9.3(range, 48 to 95)	71.1 ± 8.6 (range, 48 to 83)	76.3 ± 9.7 (range, 56 to 95)	0.121
Lens status, phakia/IOL	22/9	15/2	7/7	0.004
Anticoagulant medication (+/−)	24/7	14/3	10/4	0.671
Type of AMD (PCV/Type 1 MNV)	21/10	10/7	11/3	0.280
Duration of symptoms (days)	13.7 ± 9.3 (range, 1 to 40)	16.5 ± 10.6 (range, 4 to 40)	10.3 ± 6.2 (range, 1 to 23)	0.062
Mean size of submacular hemorrhage (DAs)	5.2 ± 1.9 (range, 3.0 to 10.7)	4.7 ± 1.3 (range, 3.0 to 6.9)	5.7 ± 2.4 (range, 3.3 to 10.7)	0.150
Mean baseline logMAR BCVA	0.78 ± 0.48	0.56 ± 0.27	1.04 ± 0.55	0.004
Mean central foveal thickness (µm)	853 ± 341	713 ± 251	1024 ± 365	0.009
Mean thickness of SMH at the fovea(µm)	522 ± 351	396 ± 195	675 ± 438	0.025
Mean thickness of hemorrhagic PED at the fovea (µm)	191 ± 321	159 ± 238	230 ± 407	0.549

VEGF = vascular endothelial growth factor; SD = standard deviation; IOL = intraocular lens; DA = disc diameter; logMAR = logarithm of the minimum angle of resolution; BCVA = best-corrected visual acuity; SMH = submacular hemorrhage; PED = pigment epithelial detachment. * *p*-value calculated using unpaired t test and the Fisher’s exact test.

**Table 2:** Multiple Regression Analysis of the BCVA after 12 months postoperatively and improvement of the visual acuity.

		Dependent Variables					
		Postoperative BCVA *			Improvement of the visual acuity **		
Independent variables		Partial Regression Coefficient	Standard Error	*p* value	Partial Regression Coefficient	Standard Error	*p* value
Lens status		0.361	0.155	0.028	0.389	0.149	0.015
Use of anticoagulants		―	―	―	−0.335	0.172	0.063
Disc areas		―	―	―	−0.054	0.040	0.191
Central foveal thickness		0.001	<0.001	0.008	0.001	<0.001	0.002
Baseline visual acuity		0.436	0.177	0.021	-0.605	0.187	0.003

BCVA = best-corrected visual acuity. * Postoperative BCVA = BCVA at 12 months after the initial treatment. ** Improvement of the visual acuity = the difference between the preoperative visual acuity and postoperative BCVA at 12 months after the treatment.

## Data Availability

The data presented in this study are available on request from the corresponding author. The data are not publicly available due to privacy or ethical restrictions.

## Abbreviations

The following abbreviations are used in this manuscript:

nAMD	neovascular age-related macular degeneration
SMH	submacular hemorrhage
PD	pneumatic displacement
tPA	tissue plasminogen activator
VEGF	vascular endothelial growth factor
VH	vitreous hemorrhage
VA	visual acuity
SD-OCT	spectral-domain optical coherence tomography
DA	disc area
RRD	rhegmatogenous retinal detachment
IVA	intravitreal injections of aflibercept
IVBr	intravitreal injections of brolucizumab
BCVA	best-corrected visual acuity
IOL	intraocular lens
CFT	central foveal thickness
PED	pigment epithelial detachment
logMAR	logarithm of the minimum angle of resolution
